# Correlated dynamics in aqueous proton diffusion†
†Electronic supplementary information (ESI) available: Oxygen–oxygen radial distribution functions for tested DFT functionals, description of bootstrapping analysis, calculated proton transfer barriers, chlorine–oxygen radial distribution functions, and hydronium–chloride correlation functions. See DOI: 10.1039/c8sc01253a


**DOI:** 10.1039/c8sc01253a

**Published:** 2018-07-30

**Authors:** Sean A. Fischer, Brett I. Dunlap, Daniel Gunlycke

**Affiliations:** a Chemistry Division , U. S. Naval Research Laboratory , Washington , DC 20375 , USA . Email: sean.fischer@nrl.navy.mil

## Abstract

The aqueous proton displays an anomalously large diffusion coefficient that is up to 7 times that of similarly sized cations. There is general consensus that the proton achieves its high diffusion through the Grotthuss mechanism, whereby protons hop from one molecule to the next. A main assumption concerning the extraction of the timescale of the Grotthuss mechanism from experimental results has been that, on average, there is an equal probability for the proton to hop to any of its neighboring water molecules. Herein, we present *ab initio* simulations that show this assumption is not generally valid. Specifically, we observe that there is an increased probability for the proton to revert back to its previous location. These correlations indicate that the interpretation of the experimental results need to be re-examined and suggest that the timescale of the Grotthuss mechanism is significantly shorter than was previously thought.

## Abstract 2

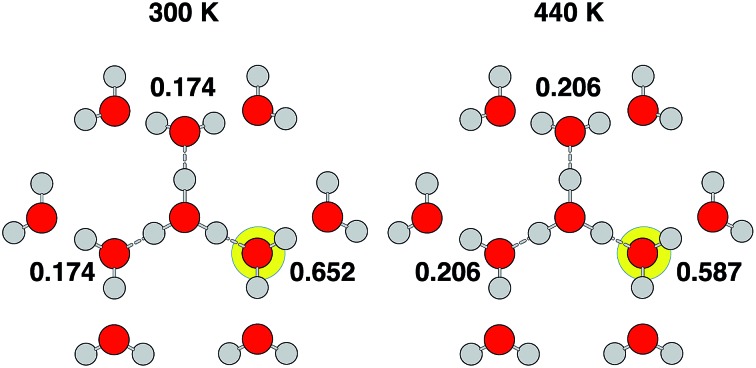
Correlated hopping directions are observed in *ab initio* simulation of proton diffusion indicating that the simple random walk model is not valid for the interpretation of experimental results.

## 1 Introduction

Transport of a proton through water is widely held to consist of two complementary processes: structural and vehicular diffusion. Structural diffusion occurs through the Grotthuss mechanism and gives the proton its large diffusion coefficient. The Grotthuss mechanism consists of shuttling protons through the hydrogen bond network of water. In between exchanges of the excess proton from one water molecule to the next, the total diffusion is supplemented by vehicular diffusion, which refers to the center-of-mass motion of the cation. A multitude of work has gone into understanding the details of the Grotthuss mechanism.^1^–^4^ Perhaps the most consistent picture of the Grotthuss mechanism is that of a generalized Eigen cation (H_9_O_4_^+^) whose central hydronium ion (H_3_O^+^) performs a “special pair dance” with the surrounding water molecules until a proton hop occurs and another molecule becomes the central hydronium ion.^5^

While the understanding of the details of the Grotthuss mechanism has evolved over time, one aspect that has remained essentially constant is the reported timescale of the Grotthuss mechanism. Since the pioneering nuclear magnetic resonance study of Meiboom,^6^ the timescale of the Grotthuss mechanism has widely been quoted as approximately 1.5 ps. Meiboom's NMR derived timescale was reinforced by the fact that a similar timescale is obtained when the structural component of the proton diffusion coefficient is modeled as a simple random walk.^6^,^7^ However, this agreement should not be too surprising as the simple random walk model was also used in order to relate the measured nuclear spin relaxation rate to the timescale of the Grotthuss mechanism.^6^,^8^

This is not to say that all studies of the Grotthuss mechanism have relied on the assumptions behind a simple random walk in performing analyses, indeed many have not.^5^,^9^–^18^ For example, some have suggested that there could be correlation in the Grotthuss mechanism *via* concerted hops along water wires.^17^–^21^ If true, this would invalidate the simple random walk model for the Grotthuss mechanism; however, they did not explore the consequences of concerted hops for the interpretation of the experimental results. Additionally, we note that other work has questioned the importance of concerted proton hops.^15^,^16^ These questions stem from the ambiguity in defining concerted hops. Voth and co-workers showed that the choice of timescale over which proton hops are considered concerted can significantly change the number of concerted hops observed.^15^,^16^ They also found that the number of concerted hops was sensitive to the chosen density functional theory exchange-correlation functional and suggested that the glassy nature of water resulting from commonly used functionals could be over emphasizing concerted hops.^15^

Of special note is a study by Halle and Karlström.^8^ They employed the idea of a correlated random walk to re-examine the connection between the measured nuclear spin relaxation rate and the timescale of the Grotthuss mechanism. While they derived a model to relate the experimental relaxation rate to the hopping timescale as a function of the degree of correlation, their work was motivated by physical arguments rather than evidence of correlation and appears not to have gained favor in the literature as judged by the lack of attention their model has subsequently received. In the end, the experimental timescale for the Grotthuss mechanism has continued to be given as approximately 1.5 ps.

In the present work, we have performed *ab initio* molecular dynamics simulations to address whether the simple random walk model is generally valid for the Grotthuss mechanism. In doing so, we have also provided one of the most statistically robust, *ab initio* determinations of the proton diffusion coefficient to date. Our simulations clearly show correlations between proton hopping directions, suggesting that the simple random walk picture is not universally valid for the Grotthuss mechanism. Consequently, the interpretation of the experimental results for the timescale of the Grotthuss mechanism should be re-examined, with our results suggesting a substantially faster hopping time.

## 2 Methods

For our molecular dynamics simulations our system consisted of 31 water molecules and one hydrochloric acid (HCl) molecule in a cubic box with an edge length of 9.87 Å. All calculations were performed with Quantum Espresso v5.4 using the CP module.^22^ The electronic structure was described by the PBE exchange-correlation functional in conjunction with ultrasoft pseudopotentials with 25 and 200 Ry cutoffs for the wave functions and charge density, respectively.^23^–^26^ We note that while there are well known deficiencies in the ability of the PBE functional to describe liquid water,^27^ previous studies have found that the underlying mechanisms of proton diffusion show only a small dependence on the choice of functional.^9^,^10^,^17^,^18^ Of course this does not imply that all these functionals are correct, so one must look at multiple metrics to ensure that physically reasonable results are being obtained. One such additional test is the ratio of the calculated proton diffusion coefficient to the calculated water diffusion coefficient, which we report in Table 1 and discuss later in the work.

**Table 1:** Calculated proton diffusion coefficients (*D*) and standard errors (SE) in units of Å^2^ ps^–1^. The last line gives the ratio of the calculated total proton diffusion coefficient to the calculated water diffusion coefficient along with the SE. The experimental ratio is 4.05 for infinite dilution^33^

	300 K		440 K	
	*D*	SE	*D*	SE
Total	1.015	0.077	3.004	0.150
Structural	0.968	0.070	2.800	0.141
Vehicular	0.139	0.007	0.403	0.018
*D*_H^+^_/*D*_H_2_O_	23.1	1.8	5.35	0.28

A Nose–Hoover chain with 4 thermostats and characteristic frequency 140 THz was used to simulate a canonical (NVT) ensemble with a target temperature of either 300 or 440 K. For our Car–Parrinello molecular dynamics,^28^ we used a time step of 4 atomic units (∼0.097 fs) and a fictitious electron mass of 300 *m*_e_ in order to keep the propagation of the system adiabatic. Data were sampled every 10 time steps, and the first picosecond of each 8 ps trajectory was discarded for equilibration. We ran 500 independent trajectories at each temperature for a total simulation time of 8 ns. The initial configurations for each trajectory were sampled from a separate molecular dynamics simulation run with the same simulation parameters.

In order to calculate a proton diffusion coefficient, we have to define the positive charge at each point along the trajectory. There is no unique way to define molecules from a collection of atoms, and this task is even more fraught with peril for an excess charge in water.^29^ In particular, the high frequency and amplitude of oxygen–hydrogen stretching vibrations can lead to an overabundance of molecular transitions if the definitions of molecules are too simplistic. Furthermore, since we aim to gain insight into the mechanisms of proton transport, we want to avoid potentially biasing the results through the definitions of the cation.

The most common approach has been to identify a hydronium ion (H_3_O^+^) as the oxygen atom closest to three hydrogen atoms in each frame of the trajectory. Whether the positive charge is identified as the hydronium ion itself or the hydronium ion is used as a stand-in for the larger Eigen cation (H_9_O_4_^+^) is often inconsequential depending on the analysis. This definition of the positive charge is susceptible to the aforementioned vibrational dynamics causing an excess of proton hops. Previous attempts to overcome this have been to simply ignore any hop that is undone by the next hop, *i.e.* if two successive hops result in the proton being in the same location as it was initially, those hops are ignored.^9^–^11^,^16^,^18^,^30^

This phenomenon has been referred to as proton rattling and has generally been ignored *a priori* in previous analyses. However, in the context of diffusive dynamics interpreted as a random walk, the proton hopping back to its previous site is a perfectly legitimate process. In fact, assuming a simple random walk, a third of the proton hops would be expected to undo the previous hop. Therefore, it is clear that if we hope to gain insight into dynamics of the Grotthuss mechanism, we need a cation definition that does not rely on the *a priori* disregard of certain types of proton transitions in order to obtain reasonable results.

To define the protonic cation at each step, we start by assigning two hydrogen atoms to every oxygen atom based on distance. The remaining hydrogen atom, which we refer to as the excess proton, is then assigned to its closest oxygen atom. If this is the first frame of the trajectory, that hydronium ion is taken as the positive charge. If this is not the first frame, then a change of the cation only occurs if the excess proton, identified by the above process, is no longer one of the three hydrogen atoms that constituted the previous hydronium ion. By limiting the hopping in this way our definition of the cation is more robust to the “special pair dance” of the excess proton within the Eigen cation^5^ and naturally eliminates most, if not all, false transitions due to vibrational dynamics. The hydronium ion oxygen atom was used as the location of the positive charge in the analysis. To separate the contributions of the structural and vehicular diffusion to the overall proton diffusion, we also kept track of whether the movement of the positive charge from one frame to the next was due to center-of-mass motion of the hydronium ion or was due to a proton exchange. Adding the increments of each class together allows us to determine the mean-squared displacements due to the individual components.

## 3 Results and discussion

**Fig. 1:**
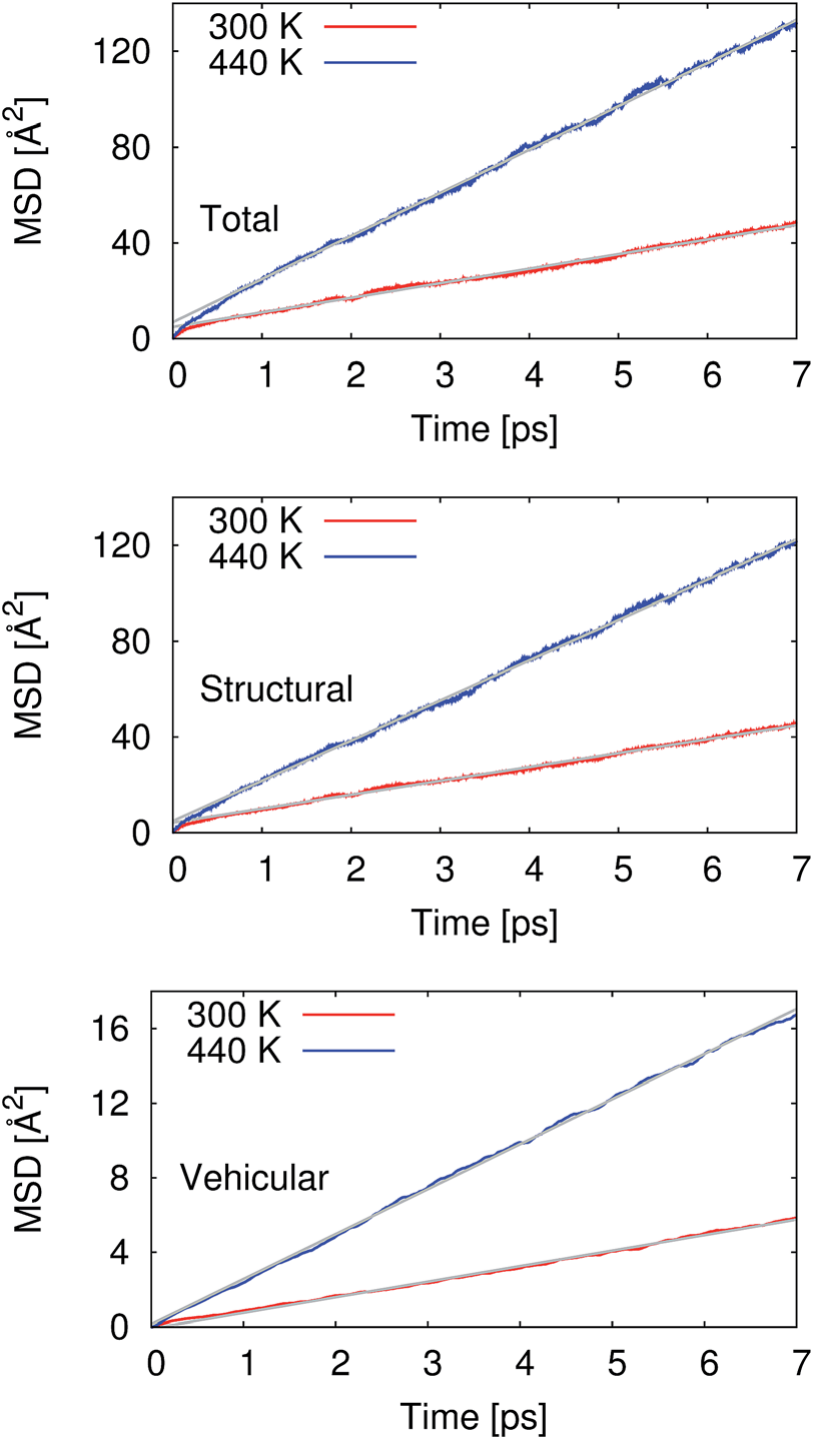
Mean-squared displacements (MSD) as functions of time for the proton at 300 and 440 K. The gray lines represent the linear regression used for extraction of the diffusion coefficients. The linear regression was performed on the data between 1 and 7 ps. The top panel gives the total MSD, while the middle and bottom panels show the structural and vehicular components, respectively.

From our molecular dynamics simulations, we calculated the mean squared displacement of the proton as a function of time. The use of hundreds of independent trajectories allows us to ensure sufficient sampling without having to resort to using overlapping trajectory segments, which bias the mean squared displacements.^31^ The mean squared displacement of the proton is shown in Fig. 1 along with the breakdown of the total into the structural and vehicular components. The diffusion coefficients were extracted from the slope of the mean squared displacement in the linear regime (between 1 and 7 ps) *via* the relation^32^1
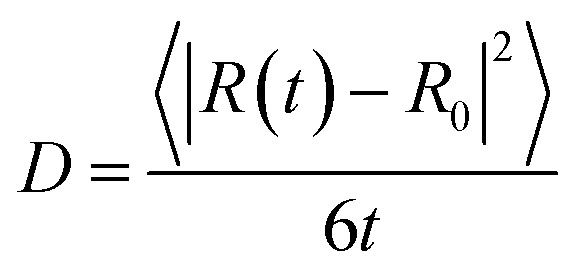
where where 〈||*R*(*t*) – *R*_0_|^2^〉 is the mean squared displacement at time is the mean squared displacement at time *t*. The resulting diffusion coefficients are collected in Table 1.

Pranami and Lamm previously showed that while linear regression can be used to obtain a point estimate of the diffusion coefficient from the mean squared displacements, the statistical uncertainty of the fitting parameters are not reflective of the uncertainty in the diffusion coefficient.^31^ To quantify the uncertainty in our calculated diffusion coefficients, we performed a bootstrapping analysis^34^,^35^ of the data set to obtain the standard errors that are also presented in Table 1. See ESI† for more details on the bootstrapping analysis.

**Fig. 2:**
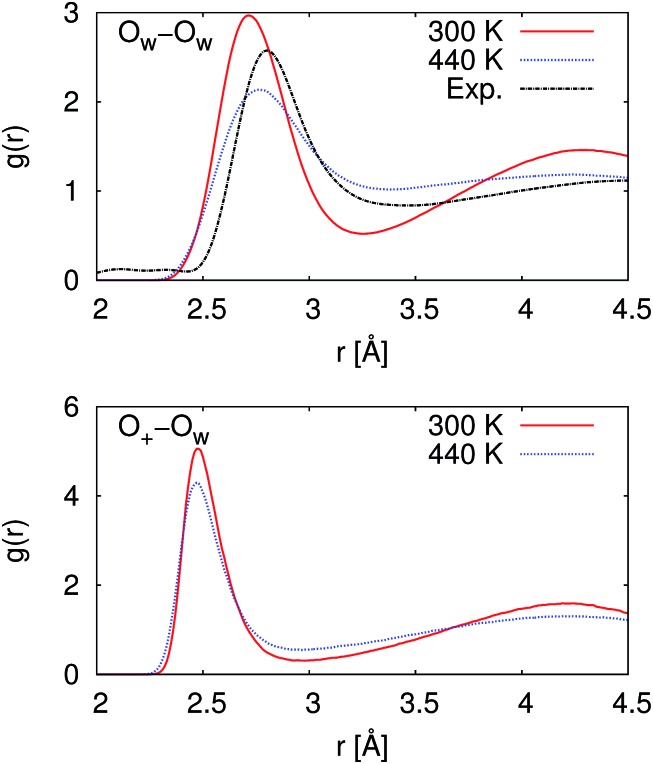
Radial distribution functions between water oxygen atoms (O_W_–O_W_) and between the hydronium ion oxygen atom and the water oxygen atoms (O_+_–O_W_). At 300 K, the simulated water is over-structured compared to the experimental reference. While at 440 K, the simulated water is now under-structured compared to the experimental reference. The experimental reference is from Skinner *et al.*,^36^ and we note that the experimental reference is for pure water while our simulations are for ∼1.7 M HCl.

At 300 K, our calculated proton diffusion coefficient of 1.015 Å^2^ ps^–1^ is close to the experimental, infinite-dilution diffusion coefficient of a proton in water at ambient conditions of 0.932 Å^2^ ps^–1^.^33^ However, our simulation setup corresponds to an acid concentration of ∼1.7 M, and the PBE exchange-correlation functional is known to over-structure water, leading to conditions more similar to super-cooled water.^27^ This is evident in the oxygen–oxygen radial distribution function shown in Fig. 2. Under these conditions, the corresponding experimental diffusion coefficient would be smaller.^37^–^39^ Additionally, as can be seen in Table 1, the ratio of the calculated proton diffusion coefficient to the calculated water diffusion coefficient is almost six times larger than the experimental ratio of 4.05 due to the glassy nature of PBE water at this temperature. That being said, our calculated proton diffusion coefficient is comparable to previously reported proton diffusion coefficients for the similarly over-structured BLYP functional (0.5 to 1.48 Å^2^ ps^–1^).^15^,^40^,^41^

In order to obtain results closer to ambient conditions, we ran a second set of simulations at 440 K, which was previously suggested as a temperature at which the PBE functional gives better liquid water properties.^27^ While real water would be a vapor at 440 K, the deficiencies in the PBE functional in describing dispersion interactions and polarizabilities result in the need to move to higher pressures and temperatures in order to simulate liquid water at the correct density.^42^ As can be seen in Fig. 2, our simulated water is now actually under-structured compared to the experimental, pure water, reference. The under-structuring is, at least partially, a result of the disruption to the water network from the excess proton and the chloride ion, as was seen in previous work on hydrochloric acid solutions.^43^,^44^ Though the description of the liquid properties of water were improved, care must still be exercised in interpreting results since it is unclear how the elevated pressure and temperature may affect other properties.

As would be expected with the increase in temperature, the calculated proton diffusion coefficient is significantly larger, 3.004 Å^2^ ps^–1^. Again, since we are dealing with a relatively concentrated system, the corresponding experimental diffusion coefficient would still be expected to be smaller than the limiting value of 0.932 Å^2^ ps^–1^, by approximately a factor 1.5.^37^ It is safe to say that PBE overestimates the proton diffusion coefficient. On the other hand, the ratio between the calculated proton and water diffusion coefficients is now in much closer agreement with experiment (Table 1). This gives some confidence that the relative dynamics are accurate even though the absolute values are overestimated.

Previous work has indicated that DFT methods underestimate the proton transfer barrier relative to wave function methods such as MP2 and CCSD(T).^45^,^46^ By proton transfer barrier, we refer to the energetic barrier to move the excess proton from one oxygen atom to a neighboring oxygen atom. A more recent study that combined coupled cluster singles and doubles (CCSD) with path-integral molecular dynamics calculated that there was no barrier to proton transfer in the protonated water dimer.^47^ Additionally, nuclear quantum effects have consistently resulted in a decreased proton transfer barrier (if one existed to begin with).^29^,^45^,^48^

Indeed, the PBE functional gives very small barriers for the proton to transfer from one oxygen atom to another (see ESI†). It is possible that the small proton transfer barrier could be the origin of the overestimated diffusion coefficients; however, the proton transfer barrier is not regarded as the rate limiting step for proton diffusion: hydrogen bond dynamics to solvating water molecules are believed to control proton diffusion.^7^,^9^,^11^,^14^,^49^,^50^ Additionally, though we only have two data points that are widely spaced, the temperature dependence of our calculated diffusion coefficients is compatible with the experimental activation energy for proton diffusion of 2–3 kcal mol^–1^ despite the proton transfer barrier being much smaller.

The calculated vehicular components to the proton diffusion coefficients are interesting in that at 300 K the vehicular diffusivity is larger than our calculated water diffusion coefficient [0.044 (SE = 0.001) Å^2^ ps^–1^], yet at 440 K the vehicular component is smaller than the calculated water diffusion coefficient [0.562 (SE = 0.007) Å^2^ ps^–1^]. The most likely explanation is that at 300 K when the water is over-structured, the water molecules are hindered in their diffusion. At the same time, the “special pair dance” of the proton causes an elevated diffusion as the central hydronium ion rattles around within its first solvation shell.^5^ When the temperature is elevated and the water molecules are more dynamic, the contribution of the “special pair dance” is not as prominent.

A common assumption concerning the proton diffusion coefficient has been that the structural and vehicular components are independent, *i.e.* structural plus vehicular equals total. Our current results suggest that this is not the case. While the differences between the sums of the components and the totals are small (0.092 at 300 K and 0.199 at 440 K), our bootstrapping analysis indicates that these differences are statistically significant at the 95% confidence level. This type of correlation between the components of the diffusion process has been suggested before based on physical arguments surrounding the polarization resulting from the hopping of the charge from one site to another.^8^ In that study, the correlation was estimated to be of the order of the vehicular component of diffusion, in agreement with our current simulations. Additionally, the same type of correlation was found previously by Xu *et al.* in their the self-consistent iterative multistate empirical valence bond simulations of HCl solutions.^51^

While correlation between the components of the diffusion process is noteworthy, it does not have any bearing on the validity of the simple random walk assumption for interpretation of the experimental results. For the simple random walk picture to be valid for the Grotthuss mechanism, the probability for the proton to hop to any of its three neighbors should be equal and not depend on the proton's history. Table 2 shows the calculated probabilities for the proton to hop back to its previous site. Our simulations clearly reveal that there is a strong preference to return to the previous site as opposed to continuing on to a new site. Though decreased slightly at the elevated temperature, the correlation is robust, suggesting that a simple random walk is not an adequate model for the Grotthuss mechanism.

**Table 2:** Calculated probabilities for the proton to return to its previous site. The standard errors of the calculated probabilities are given in the third column. For a simple random walk, the return probability would be 1/3

Simulation	Return probability	SE
PBE (300 K)	0.652	0.004
PBE (440 K)	0.587	0.003
*PBE	0.708	0.033
*PBE-D2	0.630	0.042
*revPBE	0.654	0.029
*BLYP	0.635	0.032
*BLYP-D2	0.689	0.029

In Table 2 we additionally show that the return probability is robust to the chosen exchange-correlation functional. The last five rows in Table 2 (those marked with an asterisk) are from additional simulations run to test the dependence of our results on the employed functional (PBE,^23^ PBE-D2,^52^,^53^ revPBE,^23^,^54^ BLYP,^55^,^56^ and BLYP-D2 52,53,55,56). These test simulations used the same setup as our main simulations, except we doubled the wave function and charge density cutoffs to 50 and 400 Ry, respectively. For each functional, we ran 10 trajectories in the NVT ensemble at 300 K for 8 ps. This set of functionals cover different levels of water structuring as shown in the ESI.† Despite the different descriptions of the degree of structure in the water network, all of the functionals give very similar hopping probabilities. This is inline with previous work that found the details of the Grotthuss mechanism did not show significant functional dependence.^9^,^10^,^17^,^18^

As with previous studies, we observed multiple partner exchanges as part of the “special pair dance” before the majority of the proton hopping events.^5^ This lends extra support to the enhanced return probabilities being an actual feature of the dynamics rather than an artifact of the definition of the hydronium ion. In regards to the “burst and rest” behavior seen in previous simulations,^15^,^17^ our individual trajectories are too short to observe distinct regimes that could be classified in the same manner as the other studies. However, entire trajectories could be classified as such, with some trajectories showing very little net displacement of the proton and others showing substantial displacement. That being said, we still observe normal diffusion behavior (*i.e.* a mean-squared displacement that grows linearly with time), and the calculated distribution of hopping probabilities is approximately normal, suggesting that there are not two distinct populations from which the dynamics emerge.

The implications of needing to go beyond the simple random walk model for interpreting the experimental results can be substantial. The details of the relationship between a simple random walk and the corresponding correlated random walk are known.^57^ The most relevant result is that the mean squared displacement of a correlated random walk is related to the mean squared displacement of the simple random walk by a ratio of probabilities2
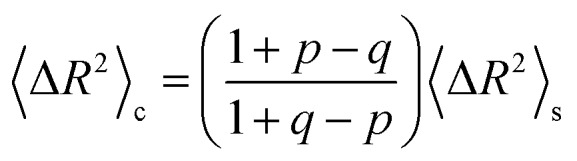
where where 〈ΔΔ*R*^2^〉_c_ represents the mean squared displacement of the correlated walk, represents the mean squared displacement of the correlated walk, 〈ΔΔ*R*^2^〉_s_ represents the mean squared displacement of the simple walk, *q* is the probability to reverse the previous hop, and *p* is the probability to hop to one of the other sites. Assuming an average hop length *ε* and hopping time *τ*, we can relate the diffusion coefficient to these quantities using the general random walk model3
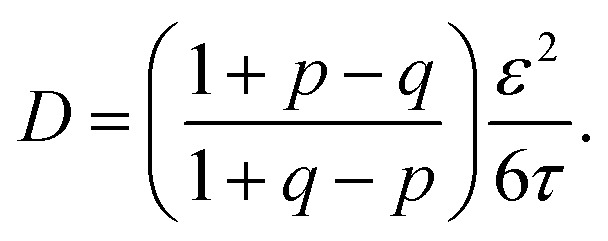



For the hydronium ion with three neighbors, we have that 2*p* + *q* = 1.

As given in Table 2, our simulations give *q* = 0.652 at 300 K and *q* = 0.587 at 440 K, both significantly different than the 1/3 expected for a simple random walk. From eqn (3), using the equilibrium distance between the hydronium ion and a water molecule of 2.5 Å (see Fig. 2), along with our calculated hopping probabilities and the experimental diffusion coefficient of 0.932 Å^2^ ps^–1^, we get a hopping timescale of between 0.460 and 0.665 ps, depending on the assumed vehicular component (sodium ion, 0.133 Å^2^ ps^–1^; water molecule, 0.230 Å^2^ ps^–1^ 33 and which set of probabilities is used. Clearly this range is substantially different from the 1.304 to 1.484 ps that is obtained from assuming a simple random walk. Note also that here we have not taken into account the correlation between the components that we found in our simulation. Doing so could lead to even faster timescales as the anti-correlation we found implies that the structural component could be even larger than what has been assumed for the experimental case. The NMR results can be reinterpreted in a similar manner.^8^

Given that our simulations were performed at an HCl concentration of ∼1.7 M, it is reasonable to wonder whether the correlations we observe can be applied to the infinite-dilution case. While a more complete answer to that question would require additional investigations, we note that the concentration of our system sits right on the edge of where changes begin to occur in the experimental vibrational spectra.^58^ In our simulations we do find that the chloride ion and hydronium ion occasionally form contact ion pairs (see ESI†), as has been seen before,^44^,^51^ and we observe some relatively long-lived correlations in the vector connecting the hydronium and chloride ions (see ESI†). Xu *et al.* did observe that the proton hopping rates decreased much more slowly than the diffusion coefficient with increases in concentration in their multistate empirical valence bond simulations.^51^ This could imply an increase in hops that undo the previous ones, though they did not calculate the relevant probabilities to know for sure. Overall, we cannot rule out ion–ion interactions leading to some of the correlation that we observe in the hopping directions.

However, we note that spectroscopic studies aiming to gain insight into aqueous proton dynamics have been done at even higher concentrations than we have studied here,^59^–^63^ providing obvious relevance for having a reliable model for the dynamics of protons in more concentrated solutions. The spectroscopy results appear to set upper and lower bounds of 2.5 ps and 480 fs for the proton transfer, and Tokmakoff and co-workers estimate that the transfer occurs in 1–2 ps.^63^ Unfortunately, none of these studies reported diffusion coefficients for their samples, which complicates interpretation of the results given that the diffusion coefficient is sensitive to concentration. Additionally, a recent theoretical study by Napoli *et al.* concluded that the dynamics measured in the spectroscopy experiments correspond to changes in the proton solvation asymmetry rather than directly relating to the proton transfer.^64^ Given the above uncertainties, it appears that the modification of the timescale due to correlation still fits within the more recent experimental results.

## 4 Conclusions

Through large sets of *ab initio* molecular dynamics simulations, we have found significant correlation between hopping directions in the Grotthuss mechanism of aqueous proton diffusion. Specifically, we found an elevated probability for the proton to return to its previous site compared to what would be expected for a simple random walk. These results suggest that the interpretation of the experimental results for proton diffusion needs to be re-examined. Until now, the experimental results have generally been interpreted in terms of a simple random walk, resulting in a timescale of approximately 1.5 ps for the Grotthuss mechanism. However, re-interpreting those results in terms of the correlated random walk suggested by our simulations, results in the timescale being closer to 0.5 ps. Furthermore, our results also provide evidence of correlation between the components of the diffusion coefficient. This could mean that the timescale of the Grotthuss mechanism is even faster since we found a negative correlation, meaning that the individual components add to more than the total. While we have found that the correlations between the components of the diffusion and the hopping directions are robust to temperature and chosen functional, further work is needed to assess the dependence of these correlations on concentration.

## Conflicts of interest

There are no conflicts to declare.

## Supplementary Material

Supplementary information

## References

[cit1] Cukierman S. (2006). Biochim. Biophys. Acta.

[cit2] Marx D. (2006). ChemPhysChem.

[cit3] Knight C., Voth G. A. (2012). Acc. Chem. Res..

[cit4] Agmon N., Bakker H. J., Campen R. K., Henchman R. H., Pohl P., Roke S., Thämer M., Hassanali A. (2016). Chem. Rev..

[cit5] Markovitch O., Chen H., Izvekov S., Paesani F., Voth G. A., Agmon N. (2008). J. Phys. Chem. B.

[cit6] Meiboom S. (1961). J. Chem. Phys..

[cit7] Agmon N. (1995). Chem. Phys. Lett..

[cit8] Halle B., Karlström G. (1983). J. Chem. Soc., Faraday Trans. 2.

[cit9] Chandra A., Tuckerman M. E., Marx D. (2007). Phys. Rev. Lett..

[cit10] Tuckerman M. E., Chandra A., Marx D. (2010). J. Chem. Phys..

[cit11] Berkelbach T. C., Lee H.-S., Tuckerman M. E. (2009). Phys. Rev. Lett..

[cit12] Crespo Y., Hassanali A. (2015). J. Phys. Chem. Lett..

[cit13] Schmitt U. W., Voth G. A. (1999). J. Chem. Phys..

[cit14] Lapid H., Agmon N., Petersen M. K., Voth G. A. (2005). J. Chem. Phys..

[cit15] Tse Y.-L. S., Knight C., Voth G. A. (2015). J. Chem. Phys..

[cit16] Chen C., Arntsen C., Voth G. A. (2017). J. Chem. Phys..

[cit17] Hassanali A., Giberti F., Cuny J., Kühne T. D., Parrinello M. (2013). Proc. Natl. Acad. Sci. U.S.A..

[cit18] Chen M., Zheng L., Santra B., Ko H.-Y., DiStasio Jr R. A., Klein M. L., Car R., Wu X. (2018). Nat. Chem..

[cit19] Hassanali A. A., Giberti F., Sosso G. C., Parrinello M. (2014). Chem. Phys. Lett..

[cit20] Cuny J., Hassanali A. A. (2014). J. Phys. Chem. B.

[cit21] Giberti F., Hassanali A. A. (2017). J. Chem. Phys..

[cit22] Giannozzi P., Baroni S., Bonini N., Calandra M., Car R., Cavazzoni C., Ceresoli D., Chiarotti G. L., Cococcioni M., Dabo I., Dal Corso A., de Gironcoli S., Fabris S., Fratesi G., Gebauer R., Gerstmann U., Gougoussis C., Kokalj A., Lazzeri M., Martin-Samos L., Marzari N., Mauri F., Mazzarello R., Paolini S., Pasquarello A., Paulatto L., Sbraccia C., Scandolo S., Sclauzero G., Seitsonen A. P., Smogunov A., Umari P., Wentzcovitch R. M. (2009). J. Phys.: Condens. Matter.

[cit23] Perdew J. P., Burke K., Ernzerhof M. (1996). Phys. Rev. Lett..

[cit24] Vanderbilt D. (1990). Phys. Rev. B.

[cit25] Laasonen K., Car R., Lee C., Vanderbilt D. (1991). Phys. Rev. B.

[cit26] Laasonen K., Pasquarello A., Car R., Lee C., Vanderbilt D. (1993). Phys. Rev. B.

[cit27] Yoo S., Zeng X. C., Xantheas S. S. (2009). J. Chem. Phys..

[cit28] Car R., Parrinello M. (1985). Phys. Rev. Lett..

[cit29] Marx D., Tuckerman M. E., Hutter J., Parrinello M. (1999). Nature.

[cit30] Wu Y., Chen H., Wang F., Paesani F., Voth G. A. (2008). J. Phys. Chem. B.

[cit31] Pranami G., Lamm M. H. (2015). J. Chem. Theory Comput..

[cit32] McQuarrie D. A., Statistical Dynamics, University Science Books, Sausalito, CA, 2000.

[cit33] Mills R. and Lobo V. M. M., Self-Diffusion in Electrolyte Solutions: A Critical Examination of Data Compiled from the Literature, Elsevier, New York, NY, 1989, vol. 36.

[cit34] Efron B. (1979). Ann. Stat..

[cit35] Efron B. (1981). Can. J. Stat..

[cit36] Skinner L. B., Huang C., Schlesinger D., Pettersson L. G. M., Nilsson A., Benmore C. J. (2013). J. Chem. Phys..

[cit37] Owen B. B., Sweeton F. H. (1941). J. Am. Chem. Soc..

[cit38] Cornish B. D., Speedy R. J. (1984). J. Phys. Chem..

[cit39] Kreuer K. D. (1996). Chem. Mater..

[cit40] Izvekov S., Voth G. A. (2005). J. Chem. Phys..

[cit41] Kabbe G., Dreßler C., Sebastiani D. (2017). Phys. Chem. Chem. Phys..

[cit42] Willow S. Y., Zeng X. C., Xantheas S. S., Kim K. S., Hirata S. (2016). J. Phys. Chem. Lett..

[cit43] Botti A., Bruni F., Imberti S., Ricci M. A., Soper A. K. (2004). J. Chem. Phys..

[cit44] Heuft J. M., Meijer E. J. (2006). Phys. Chem. Chem. Phys..

[cit45] Pavese M., Chawla S., Lu D., Lobaugh J., Voth G. A. (1997). J. Chem. Phys..

[cit46] Ojamäe L., Shavitt I., Singer S. J. (1998). J. Chem. Phys..

[cit47] Spura T., Elgabarty H., Kühne T. D. (2015). Phys. Chem. Chem. Phys..

[cit48] Marx D., Tuckerman M. E., Parrinello M. (2000). J. Phys.: Condens. Matter.

[cit49] Tuckerman M., Laasonen K., Sprik M., Parrinello M. (1995). J. Phys. Chem..

[cit50] Tuckerman M., Laasonen K., Sprik M., Parrinello M. (1995). J. Chem. Phys..

[cit51] Xu J., Izvekov S., Voth G. A. (2010). J. Phys. Chem. B.

[cit52] Grimme S. (2006). J. Comput. Chem..

[cit53] Barone V., Casarin M., Forrer D., Pavone M., Sambi M., Vittadini A. (2009). J. Comput. Chem..

[cit54] Zhang Y., Yang W. (1998). Phys. Rev. Lett..

[cit55] Becke A. D. (1998). Phys. Rev. A.

[cit56] Lee C., Yang W., Parr R. G. (1988). Phys. Rev. B.

[cit57] Chen A., Renshaw E. (1994). J. Appl. Probab..

[cit58] Daly Jr C. A., Streacker L. M., Sun Y., Pattenaude S. R., Hassanali A. A., Petersen P. B., Corcelli S. A., Ben-Amotz D. (2017). J. Phys. Chem. Lett..

[cit59] Woutersen S., Bakker H. J. (2006). Phys. Rev. Lett..

[cit60] Decka D., Schwaab G., Havenith M. (2015). Phys. Chem. Chem. Phys..

[cit61] Thämer M., De Marco L., Ramasesha K., Mandal A., Tokmakoff A. (2015). Science.

[cit62] Biswas R., Carpenter W., Fournier J. A., Voth G. A., Tokmakoff A. (2017). J. Chem. Phys..

[cit63] Carpenter W. B., Fournier J. A., Lewis N. H. C., Tokmakoff A. (2018). J. Phys. Chem. B.

[cit64] Napoli J. A., Marsalek O., Markland T. E. (2018). J. Chem. Phys..

