# Supplementary material for: Correlated dynamics in aqueous proton diffusion
Source: Chem Sci. 2018 Jul 30;9(35):7126–32. doi: 10.1039/c8sc01253a (PMC6137442; doi:10.1039/c8sc01253a)
Supplement: Supplementary file 1 [file SC-009-C8SC01253A-s001.pdf]

Electronic Supplementary Material (ESI) for Chemical Science.
This journal is © The Royal Society of Chemistry 2018

Electronic supplementary information: Correlated dynamics in aqueous proton diffusion

Sean A. Fischer,* Brett I. Dunlap, Daniel Gunlycke

Chemistry Division, U. S. Naval Research Laboratory,
Washington, DC 20375, USA

*To whom correspondence should be addressed; E-mail: sean.fischer@nrl.navy.mil.

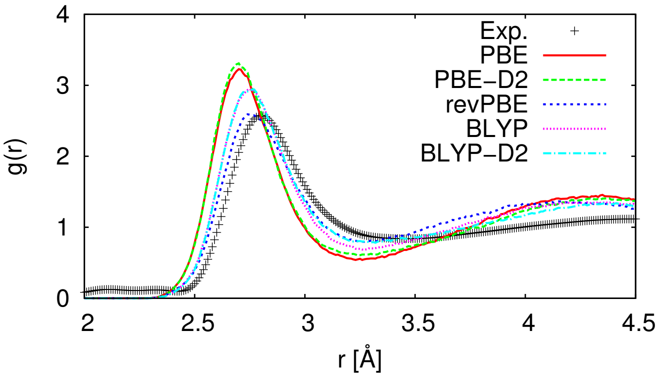

Figure S1: Oxygen-oxygen radial distribution functions for the tested functionals and the experimental results of Skinner et al.[1]

Displayed in Fig. S1 are the water oxygen-oxygen radial distribution functions for the additional test calculations we performed to test the dependence of the observed correlation on the DFT approximation.

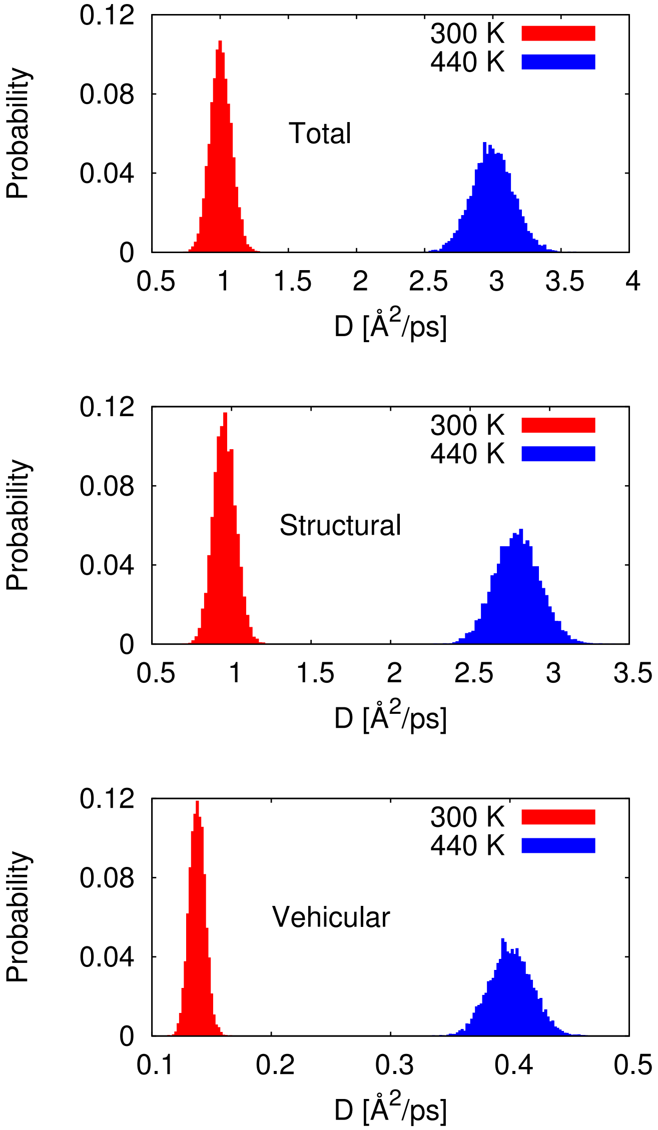

Figure S2: Distribution of the diffusion coefficients calculated in the bootstrapping analysis.

Because the fitting parameters from the linear regression of the mean squared displacements are not representative of the uncertainty in the calculated diffusion coefficients,[2] we performed a bootstrapping analysis in order to quantify our uncertainty.[3,4] Bootstrapping is a resampling technique where random sampling with

replacement is used to assign measures of accuracy to sample estimates. For the present case, our set of 500 trajectories for each temperature served as our sample. We randomly selected with replacement 500 of those trajectories to generate a new mean squared displacement as a function of time, which was then used to calculated a new diffusion coefficient. This was repeated 10,000 times for each temperature. The standard deviations of the bootstrap distributions are the estimates of the standard errors of the diffusion coefficients.[5] The distributions of the diffusion coefficients calculated from the bootstrapping analysis are shown in Fig. S2.

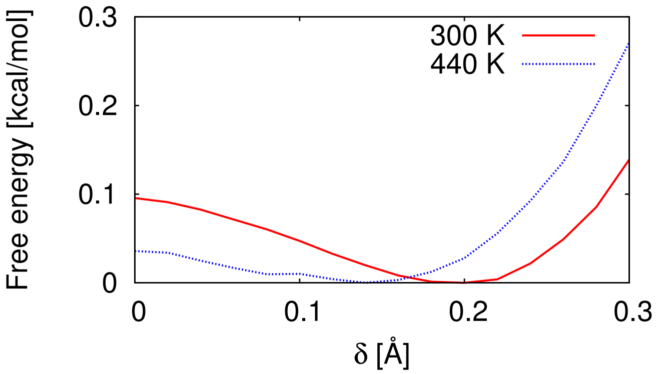

Figure S3: Calculated free energy for the proton to move from one oxygen atom to another as a function of the proton sharing coordinate.

Figure S3 shows the calculated proton transfer barriers from our molecular dynamics simulations. The proton sharing coordinate δ in Fig. S3 is defined as the difference between the distance from one oxygen atom to the proton and the distance between a second oxygen atom and the proton. A value of zero for this coordinate corresponds to the proton being in the middle of the two oxygen atoms. For reference, thermal energy ($k_B T$) is 0.596 kcal/mol at 300 K and 0.874 kcal/mol at 440 K.

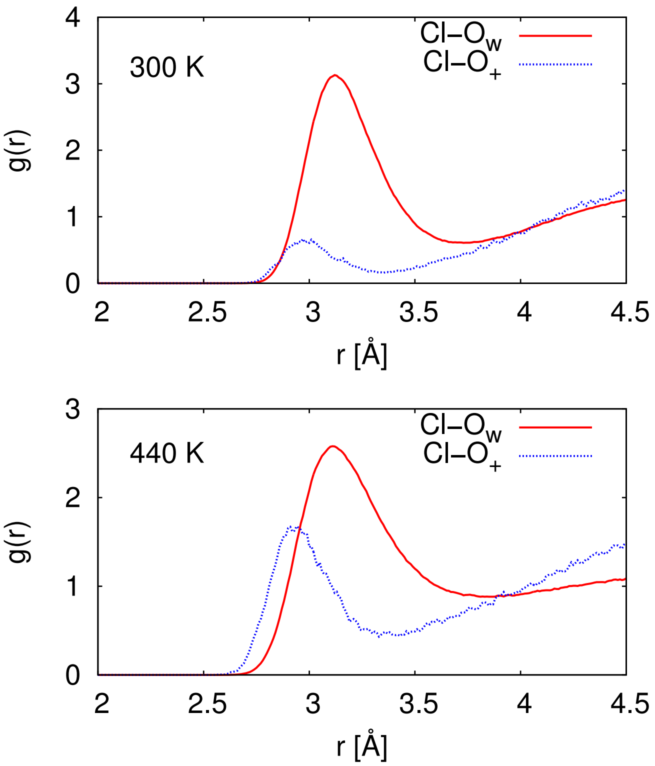

Figure S4: Radial distribution functions between the chloride ion and water oxygen atoms (Cl-O_W) and between the chloride ion and the hydronium ion oxygen atom (Cl-O_+).

Figure S4 shows the radial distribution function between the chloride ion and the water oxygen atoms (O_W), and between the chloride ion and the hydronium oxygen atoms (O_+). The peak around 3 Å is due to oxygen atoms in the first solvation shell of the chloride ion, indicating that contact ion pairs are occasionally formed between the hydronium and chloride ions.

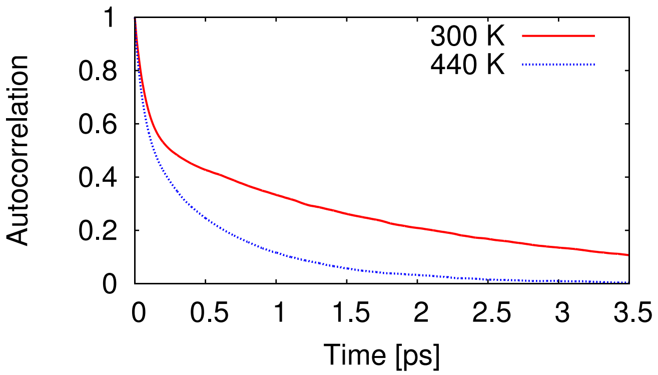

Figure S5: Normalized autocorrelation for the vector connecting the hydronium and chloride ions.

Figure S5 shows the autocorrelation function of the vector connecting the hydronium and chloride ions. Though the hydronium and chloride ions form contact pairs more often at 440 K, the correlation between the two ions decays more quickly at higher temperature.

References

[1] L. B. Skinner, C. Huang, D. Schlesinger, L. G. M. Pettersson, A. Nilsson, and C. J. Benmore. Benchmark oxygen-oxygen pair-distribution function of ambient water from x-ray diffraction measurements with a wide Q-range. *J. Chem. Phys.*, 138:074506, 2013.

[2] G. Pranami and M. H. Lamm. Estimating error in diffusion coefficients derived from molecular dynamics simulations. *J. Chem. Theory Comput.*, 11:4586–4592, 2015.

[3] B. Efron. Bootstrap methods: Another look at the jackknife. *Ann. Stat.*, 7:1–26, 1979.

[4] B. Efron. Nonparametric standard errors and confidence intervals. *Can. J. Statist.*, 9:139–172, 1981.

[5] B. Efron and R. Tibshirani. Bootstrap methods for standard errors, confidence intervals, and other measures of statistical accuracy. *Stat. Sci.*, 1:54–77, 1986.
